# The identification of novel Mycobacterium tuberculosis DHFR inhibitors and the investigation of their binding preferences by using molecular modelling

**DOI:** 10.1038/srep15328

**Published:** 2015-10-16

**Authors:** Wei Hong, Yu Wang, Zhe Chang, Yanhui Yang, Jing Pu, Tao Sun, Sargit Kaur, James C. Sacchettini, Hunmin Jung, Wee Lin Wong, Lee Fah Yap, Yun Fong Ngeow, Ian C. Paterson, Hao Wang

**Affiliations:** 1 School of Chemistry and Chemical Engineering, Beifang University of Nationalities, Yinchuan, 750021, P. R. China; 2 School of Pharmacy, Ningxia Medical University, Yinchuan, 750004, P. R. China; 3 School of Basic Medicine, Ningxia Medical University, Yinchuan, 750004, P. R. China; 4 Key Laboratory of Fertility Preservation and Maintenance of Ministry of Education, Ningxia Medical University, Yinchuan, 750004, P.R. China; 5 Department of Pre-Clinical Sciences, Faculty of Medicine and Health Sciences, Universiti Tunku Abdul Rahman, Bandar Sungei Long, 43000, Malaysia; 6 Department of Biochemistry and Biophysics, Texas A & M University, College Station, TX 77843, USA; 7 Department of Oral Biology and Biomedical Sciences and Oral Cancer Research and Coordinating Centre, Faculty of Dentistry, University of Malaya, Kuala Lumpur, 50603, Malaysia

## Abstract

It is an urgent need to develop new drugs for *Mycobacterium tuberculosis* (Mtb), and the enzyme, dihydrofolate reductase (DHFR) is a recognised drug target. The crystal structures of methotrexate binding to *mt*- and *h*-DHFR separately indicate that the glycerol (GOL) binding site is likely to be critical for the function of *mt*-DHFR selective inhibitors. We have used *in silico* methods to screen NCI small molecule database and a group of related compounds were obtained that inhibit *mt*-DHFR activity and showed bactericidal effects against a test Mtb strain. The binding poses were then analysed and the influence of GOL binding site was studied by using molecular modelling. By comparing the chemical structures, 4 compounds that might be able to occupy the GOL binding site were identified. However, these compounds contain large hydrophobic side chains. As the GOL binding site is more hydrophilic, molecular modelling indicated that these compounds were failed to occupy the GOL site. The most potent inhibitor (compound **6**) demonstrated limited selectivity for *mt*-DHFR, but did contain a novel central core (7H-pyrrolo[3,2-f]quinazoline-1,3-diamine), which may significantly expand the chemical space of novel *mt*-DHFR inhibitors. Collectively, these observations will inform future medicinal chemistry efforts to improve the selectivity of compounds against *mt*-DHFR.

Tuberculosis (TB) is a deadly infectious disease caused by *Mycobacterium tuberculosis* (Mtb) that is particularly prevalent in South-East Asia and Africa. In 2013, it is estimated that 9 million people developed TB and 1.5 million died from the disease^1^. Despite the fact that death from TB is often preventable, the rapid increase of multidrug-resistant tuberculosis and extensively drug-resistant tuberculosis has resulted in an urgent need to develop new drug targets for Mtb^2^,^3^.

The enzyme, dihydrofolate reductase (DHFR), catalyzes NADPH-dependent reduction of dihydrofolate to tetrahydrofolate, which is a precursor of cofactors necessary for the synthesis of thymidylate, purine nucleotides, methionine, serine, and glycine that are required for DNA, RNA, and protein synthesis^4^,^5^. Specific inhibitors of mycobacterial DHFR (*mt*-DHFR) that are active against live Mtb cells have been developed, suggesting that such inhibitors may be useful for treating TB^6^. Highly potent inhibition of DHFR has been achieved with analogues of the substrate, dihydrofolate, and one of the most well-known inhibitors is Methotrexate (MTX, Fig. 1), which binds to both human DHFR (*h*-DHFR) and *mt*-DHFR without any significant selectivity^7^,^8^. Such inhibitors contain 2,4-Diaminopteridin as the central core and are referred to as classical inhibitors. However, the full pteridinediamine structure is not required and non-classical inhibitors were subsequently developed that contain pyrimidine-2,4-diamines or analogues as the central cores, such as Trimethoprim (TMP)^9^, Pyrimethamine^10^,^11^, Methylbenzoprim^12^ and Bromo-WR99210^13^,^14^ (Fig. 1).

Analysis of the crystal structures of MTX binding to *mt*- and *h*-DHFR indicate that *mt*-DHFR binds with MTX, glycerol (GOL) and NADPH (PDB ID: 1DF7) (Fig. 2)^15^, but the GOL binding site is essentially absent in *h*-DHFR (PDB ID: 1OHJ, *h*-DHFR complexed with NADPH and PT523)^16^. The *mt*-DHFR active site can be roughly divided into 2 parts, the dihydrofolate and GOL binding sites. Most DHFR inhibitors target the dihydrofolate binding site. The GOL binding site is a relative small pocket, and is very close to the dihydrofolate binding site, such that it might be treated as an extension of dihydrofolate binding site. It has been assumed that the GOL binding site is critical for the function of *mt*-DHFR selective inhibitors^15^,^17^. For example, El-Hamamsy *et al*., designed and synthesised a group of compounds containing GOL-like side chains, and among these compounds, compound **El-7a** (Fig. 1) showed notable selectivity for inhibition effects on *mt*-DHFR against *h*-DHFR^17^. However, the assay they used to evaluate the abilities of **El-7a** was based on TB5 *Saccharomyces cerevisiae* carrying the DHFR genes from M. *tuberculosis* and human. Therefore, there is no direct evidence to show **El-7a** can inhibit the growth of M. tuberculosis.

In the present study, pharmacophore models were built using *in silico* methods that considered the influence of the GOL binding site. A group of related compounds were obtained and tested for their ability to inhibit *mt*-DHFR activity and for the bactericidal effects against a test Mtb strain. Of these, a group of compounds that significantly inhibited the growth of Mtb (without strong selectivity against *h*-DHFR) were obtained. In order to understand the relatively low selectivity (5 times vs *h*-DHFR for the most potent compound), the influence of GOL binding site was studied using molecular dynamic simulations and free energy calculations, as it was believed that the GOL binding site could be important for the selectivity.

## Methods

### Selection of Docking Libraries

The National Cancer Institute compounds library (NCI Release 4, 265,242 compounds, published in May 2012) was chosen for computer screening. The 3D conformations of all the molecules were generated using the Sybyl (v 7.3; Tripos) virtual screening tools.

### System Setup

The crystal structure of *mt*-DHFR in complex with NADPH, GOL and MTX (PDB ID: 1DF7)^15^ was taken as the receptor. The bound ligand, MTX, was extracted, and used as a reference to indicate the binding site. The receptor was treated with Amber 12^18^, not only for virtual screening and molecular docking, but also for subsequent molecular dynamic simulations. The parameters of NADPH were obtained from AMBER parameter database (http://www.pharmacy.manchester.ac.uk/bryce/amber/, uploaded by U. Ryde) and the *ab initio* QM calculations were performed on GOL using the B3LYP 6-31G* basis set within Gaussian09^19^. The molecular geometries were optimised and the atom-centered point charges were calculated to fit the electrostatic potential using RESP^20^. The receptor was treated by leap module in Amber 12 with amber ff12SB (for the protein part) and Generalized Amber Force Field (GAFF, for the GOL part)^21^ force field, and then the receptor was saved in the pdb format for virtual screening and molecular docking.

### Virtual screening

**Figure 3:**
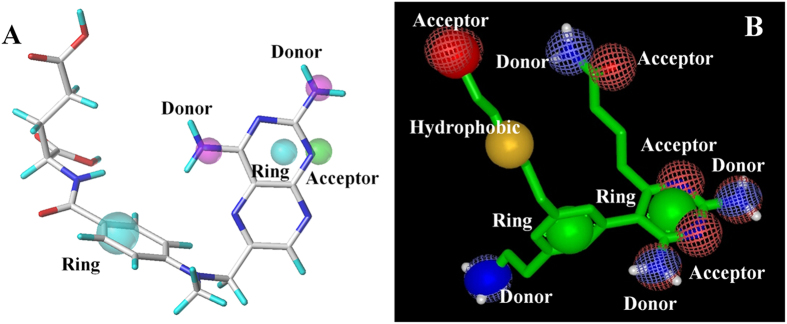
(**A**) 3D-Pharmacophore model generated by Sybyl, and MTX was aligned to the model to make it clear. (**B**) The decoy molecule created for vROCS search.

A total of 78 inhibitors of *mt*-DHFR were collected from the PDB (ligands complexed with protein) and various literature sources^17^,^22^,^23^,^24^,^25^. All these compounds were docked into *mt*-DHFR to understand their interactions with the protein. A 3D-pharmacophore model was then built and used to search the NCI database (National Cancer Institute small molecule database release 4) using the Unity module within Sybyl, as shown in Fig. 3A. As compound **El-7a**^17^ was predicted to occupy the GOL binding site which may cause the selectivity against *h*-DHFR, a decoy compound template was designed and used as a reference to perform a similarity search using vROCS (version 3.1.2, Openeye)^26^ with default settings (Fig. 3B). In the molecule template, the acylamino side chain was used to mimic the hydrogen bond acceptors and donors on GOL.

### Molecular Docking

#### FRED Docking

FRED (v 3.0.1; Fast Rigid Exhaustive Docking)^27^ is a protein-ligand docking program released by Openeye. The hits obtained from previous virtual screening were treated with the Omega2 (v 2.5.1.4)^28^ module in the Openeye package using the default settings to generate a multi-conformers molecular library, and saved at most 200 conformations per compound. The hits library was then docked to *mt*-DHFR using FRED with default settings, and ranked by the Chemgauss 4 scoring function. The top 500 compounds were recorded for further consideration.

#### GOLD Docking

GOLD (v 5.2.2 Genetic Optimization for Ligand Docking)^29^ was used to dock each ligand 10 times, starting each time from a different random population of ligand orientations and using the default automatic genetic algorithm parameter settings. All torsion angles in each compound were allowed to rotate freely and the results of the different docking runs were ranked using Gold Score. The top 500 molecules were considered for experimental testing.

### Generation of recombinant enzymes and *in vitro* assays for Mtb and human DHFR

Recom-binant DHFR enzymes were expressed and purified as described previously^30^. Enzyme assays were performed in 100 mM HEPES, 50 mM KCl, pH 7.0 at 25 °C. The absorbance decrease at 340 nm representing the oxidation of NADPH was monitored with a spectrophotometer (Cary 50, Varian, Palo Alto, CA). Inhibitors were added to a 1 ml cuvette at various concentrations, with the individual DHFRs (20 nM), and 40 μM of NADPH, and the reaction was initiated by the addition of 40 μM of dihydrofolate. For IC_50_ determinations, 20 nM of DHFRs was incubated with 40 μM of cofactor NADPH and seven serially diluted concentrations of the inhibitors for 1 minute. The reaction was initiated by the addition of 40 μM dihydrofolate. The reaction progress was measured for 2 minutes, and the linear region was used to determine the initial velocity parameters. Percent inhibition values from different concentration points were analysed by the curve fitting program supported by the Collaborative Drug Discovery.

### Microbroth culture for bacteriostatic and bactericidal activity

The anti-mycobacterial activity of the compounds was tested by the measurement of OD readings and agar plate cultures. The test strain used was a stock culture of *M. tuberculosis* H37Ra (ATCC 25177) stored at −80 °C. Prior to testing, the stock culture was thawed and grown on Middlebrook 7H10 agar (Difco, USA) to check for viability and purity. To prepare the inoculum, a suspension of the culture in Middlebrook 7H9 broth (Difco, USA) was adjusted to a turbidity equivalent to McFarland standard no.1 and further diluted to a final concentration of approximately 10^5^ cfu/ml. The compounds were dissolved in DMSO and diluted in 1% DMSO in 7H9 broth to obtain 100 μg/ml, 50 μg/ml, 10 μg/ml, 5 μg/ml 1 μg/ml and 0.1 μg/ml of compound. Each dilution was pipetted in 150 μl duplicates into a sterile 96-well microtitre plate. Three controls were set up with (a) 1% DMSO in 7H9 broth (b) 7H9 broth without DMSO and (c) p-amino salicylic acid (PAS) at 4 μg/ml (26.12 μM) in 7H9 broth with 1% DMSO. All wells were then inoculated with 10 μl of the mycobacterial culture at 10^5^ cfu/ml, sealed with parafilm and incubated at 36 °C for 28 days. OD readings (at 630 nm wavelength) were taken daily using TECAN spectrophotometer with Magellan software version 7.1. In addition, on day 14 and day 28, 10 μl was removed from each well for subculturing on compound-free 7H10 agar plates which were incubated up to six weeks at 36 °C. The number, size and time of appearance of colonies in each subculture was recorded. A random sample of colonies was stained for acid-fastness to exclude non-mycobacterial contamination. Bactericidal activity was indicated by no growth of the test strain in the subculture, up to six weeks of incubation. Compounds showing less increase in OD readings than the no-compound controls but positive growth in the subcultures were deemed to have bacteriostatic activity.

### Molecular Dynamic Simulation

Each selected compound and *mt*-DHFR complex was explicitly solvated in a truncated octahedral box of TIP3P model water (at least 12 Å from the complex to avoid periodic artifacts from occurring) by using Amber 12 with the amber ff12SB force field. Enough *Na*^+^ ions were added to neutralize the charges of the system by using the “addions” command line within the tleap module (AmberTools 12, which adds counterions around the complex using a Coulombic potential on a grid). The whole system was first optimised by energy minimisation, followed by 40 ns molecular dynamic simulation. To obtain the parameters for the ligands and GOL, QM calculations were performed by using the B3LYP 6-31G* basis set within Gaussian09 to optimise molecular geometries, and the atom-centred point charges were calculated to fit the electrostatic potential using RESP. The parameters of NADPH were obtained from the AMBER parameter database, as described previously. GOL and each ligand were treated by GAFF for parameters. We used the Principal Component Analysis (PCA) approach to check for equilibration and sampling. At least the last 20 ns stable, equilibrated trajectories of each simulation were taken for energy analysis.

The standard equilibration protocol used was as follows:

1. The solvent was energy minimised by 50 steps of the steepest descent method and then followed by 10000 steps of the conjugate gradient method with the default nonbonded cutoff of 8 Å, while the solute was held fixed.
2. The entire system was energy minimised with the same settings as the previous step.
3. The solvent was subjected to a short (20 ps) MD simulation at a temperature of 100 K, under constant pressure conditions. For this and all following simulations, MD simulations were performed with explicit solvent models and in the NPT ensemble (T = 300 K; P = 1 atm). Periodic boundary conditions (PBC) and particle-mesh-Ewald method (PME)^31^ were used to model long-range electrostatic effects, with the temperature coupled to an external bath using a weak coupling algorithm^32^. The cutoff non-bonded interaction was set as 8 Å. The bond interactions involving H-atoms were constrained by using the SHAKE algorithm.
4. Over 20 ps, the solvent temperature was raised to 300 K. During both this phase and the last, position restraints on every solute atom (force constant 100 kcal/mol/Å^2^) maintained them in their energy-minimised conformation.
5. Over a series of 20 ps constant–pressure simulations at 300 K, the restraints on the solute were gradually relaxed (50, 25, 10, 5, 2, and then 1 kcal/mol/Å^2^).
6. The final 200 ps MD simulations were performed as the equilibration runs without any restraints.

After the energy minimisation and equilibrations, production MD was run for 40 ns in an NPT ensemble at 1 atm and 300 K. The time step necessary to solve the Newton’s equations was chosen to be equal to 2 fs and the trajectory files were collected every 10 ps for the subsequent analysis. All trajectory analysis was performed with the Ptraj module in the AmberTools 12 and examined visually using VMD software^33^.

In the present work, in total 9 series of molecular simulations were performed, and they are: El-7a in complex with *mt*-DHFR in the absence (Pose 1 and 2) and in the presence (Pose 3) of GOL; compound 2 in complex with *mt*-DHFR in the absence (Pose 1, 2 and 3) and in the presence (Pose 4) of GOL; compound 6 in complex with *mt*-DHFR and in complex with *h*-DHFR.

### Binding Free Energy Calculations

The binding free energy can be calculated using equation 1 from a well-equilibrated molecular dynamics simulation:


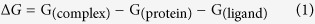


Where G is the average free energy calculated from a set of structures taken from the equilibrated simulation (snapshots). Two popular choices to calculate the binding free energy of the snapshots are the Poisson–Boltzman (PBSA) and generalised Born (GBSA) models^34^,^35^,^36^,^37^,^38^, where SA corresponds to an estimation of the non-polar solvation free energy based on a simple surface area term.

In the present study, one thousand snapshots collected from the last 20 ns stable simulations at 20 ps intervals were used, and MM-PBSA was chosen to calculate the binding free energies with the Python script, MMPBSA.py, included in AMBERTOOLS 13. The nonpolar solvation free energy (ΔG_np_) was determined by the solvent accessible surface area (SASA) according to equation 2.


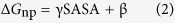


Where the surface tension γ and the offset β were set to the standard values of 0.00542 kcal/mol/Å^2^ and 0.92 kcal/mol, respectively. Other options were set to default settings. The entropy was estimated by using the Normal Mode program within the AMBER suite. Because these calculations are computationally intensive, only 100 snapshots for each MD trajectories were used for the normal-mode analysis of the equilibrated structure.

In the present work, in total 8 series of binding free energy calculations were performed, and they are: El-7a in complex with *mt*-DHFR in the absence (Pose 1 and 2) of GOL; compound 2 in complex with *mt*-DHFR in the absence (Pose 1, 2 and 3) and in the presence (Pose 4) of GOL; and compound 6 in complex with *mt*-DHFR and in complex with *h*-DHFR.

## Results and Discussion

### Virtual screening and Molecular Docking

The docking results were clustered by using Discovery Studio and then carefully analysed manually. After removal of duplicates and those molecules that have already been previously reported as *mt*-DHFR inhibitors, 213 compounds (158 molecules selected from the hit list based on vRocs searching, and 55 from Sybyl searching) were chosen from the final hit list. Following manual inspection with the combined aims of covering the maximum amount of chemical space while avoiding non drug-like function groups, 40 compounds (26 molecules based on vRocs searching, and 14 on Sybyl searching) were subsequently selected and ordered from NCI to test for their ability to inhibit *mt*-DHFR and *h*-DHFR.

### Inhibition of DHFR activities *in vitro*

The 40 compounds were tested for their ability to inhibit recombinant *mt*-DHFR and *h*-DHFR activity in *in vitro* enzyme assays. Eight compounds showed strong inhibition effects on *mt*-DHFR and three of these compounds (6, 7, and 8) were more potent inhibitors of mt-DHFR compared to h-DHFR ( Table 1).

### Antibacterial activity

In the microbroth cultures for the demonstration of mycobacterial inhibition, the 7H9 broth and 1% DMSO controls showed a steady increase in OD readings and yielded abundant growth of *M. tuberculosis* H37Ra colonies within 14 days of incubation on 7H10 agar. In the presence of para-aminosalicylic acid (PAS), one of the standard anti-TB drugs, there was only scanty growth plus a two-week delay in the appearance of colonies. These results are consistent with uninhibited mycobacterial multiplication in 1% DMSO and a more than two-log reduction in growth in the presence of PAS. All eight compounds showed varying degrees of growth inhibition on the test strain, seen as total inhibition of growth, reduction in colony counts, delayed appearance of colonies or slower increase in colony size. The final colony counts after four weeks of exposure to the compounds are shown in Table 2. At 100μg/ml, three compounds (**4**, **6** and **8**) showed bactericidal activity. At 50 μg/ml, only one (compound **6**) was still bactericidal, while at 10 μg/ml, only compound **6** (at 25.37 μM) showed a notable reduction in colony count. Compound **7** showed the least growth inhibition, affecting the colony size but not the colony count. After exposure to 50 μg/ml of compound **8**, the test strain did not grow in the 14-day subculture but formed abundant colonies in the 28-day subculture. This unexpected finding raised the possibility of induced resistance in which small numbers of mutants, when given a selective advantage in the presence of the compound, multiplied to become a predominantly resistant population. Overall, at 100 μg/ml, compounds **4**, **6** and **8** showed better activity than PAS; at 50 μg/ml, compounds **2**, **3** and **4** showed similar activity as PAS; but at 10 μg/ml, only compound **6** (25.37 μM) retained the same activity as PAS (4 μg/ml, 26.12 μM).

### Molecular Dynamic Simulation

#### The influence of GOL on the binding of compound El-7a

**Figure 4:**
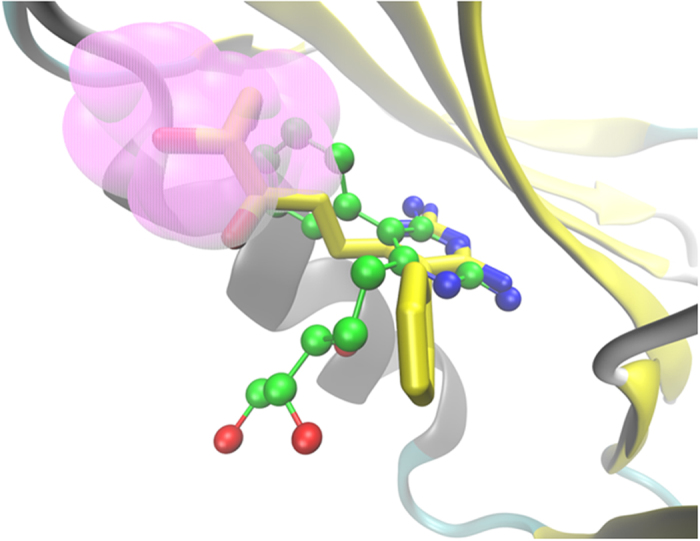
The alignment of compound **El-7a** in binding poses 1 and 2 in the system in the absence of GOL. The carbon atoms of compound **El-7a** in pose 1 are yellow, and those in pose 2 are shown in green. The GOL binding site is indicated in pink.

We were interested to examine the influence of GOL in the binding of potential *mt*-DHFR inhibitors because the GOL binding site is essentially absent in *h*-DHFR (PDB ID: 1OHJ, *h*-DHFR complexed with NADPH and PT523)^10^. Therefore, the binding poses of compound **El-7a** were analysed in the absence or presence of GOL. In the absence of GOL, compound **El-7a** can be docked with two binding poses (Pose 1, the propanetriol group occupied the GOL site; Pose 2, the propanetriol group pointed to the opposite direction of GOL site, Fig. 4). In the presence of GOL, compound **El-7a** could only be docked with one binding pose (pose 3) (both the propanetriol group and the benzene ring pointed to the opposite direction of GOL site). Molecular dynamic simulations, MM-PBSA and Normal Mode calculations were performed on these three binding poses to generate more detailed conformational and energy information.

**Figure 5:**
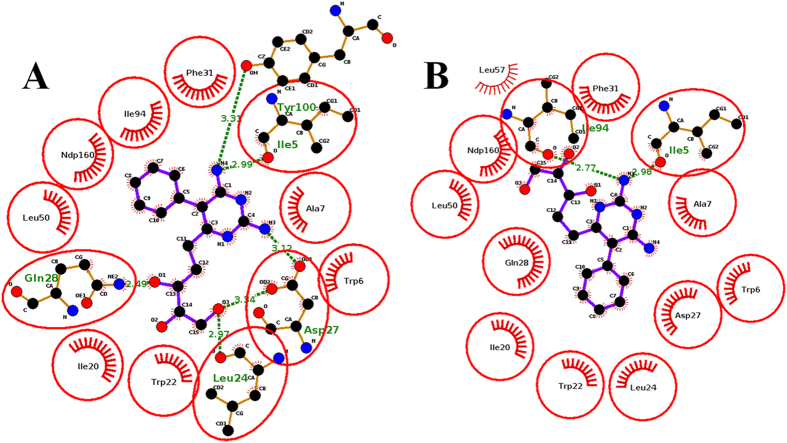
Compound **El-7a** in pose 1 (**A**) and in pose 2 (**B**). The hydrogen bonds are represented by green dotted lines.

In the absence of GOL, both pose 1 and pose 2 were stable. The average structures from the trajectories shown in Fig. 5 indicate that in pose 1 compound **El-7a** formed hydrogen bonds with Ile5, Asp27, Gln28, Leu24 and Tyr100, while in pose 2, it formed hydrogen bonds with Ile5 and Ile94. MM-PBSA and Normal Mode calculations were performed on these two simulations and the results showed the binding free energy of pose 1 was lower than that of pose 2, which indicated that pose 1 is a more stable binding pose (Table 3). This result suggests the propanetriol group of compound **El-7a** may occupy the GOL binding site. The free energy decomposition of MM-PBSA calculation was performed to analyze the interaction of **El-7a** with the active site in Pose 1. We observed that 6 residues showed strong contributions to the binding free energy (lower than −1.0 kcal/mol). They are Ile5 (−1.61 Kcal/mol), Trp6 (−1.89 Kcal/mol), Ala7 (−1.36 Kcal/mol), Ile20 (−1.66 Kcal/mol), Trp22 (−2.43 Kcal/mol) and Phe31 (−1.83 Kcal/mol). Among these residues, Ile5, Trp6, Ala7 and Phe31 show strong interactions with the pyrimidine-2,4-diamines group, and Ile20 and Trp22 form interactions with the propanetriol group (GOL binding site). The major free energy contribution of Ile20 comes from the VDW interaction, whilst the major contributions of Trp22 are from both VDW (−0.95 Kcal/mol) and electrostatic (−2.41 Kcal/mol) interactions.

**Figure 6:**
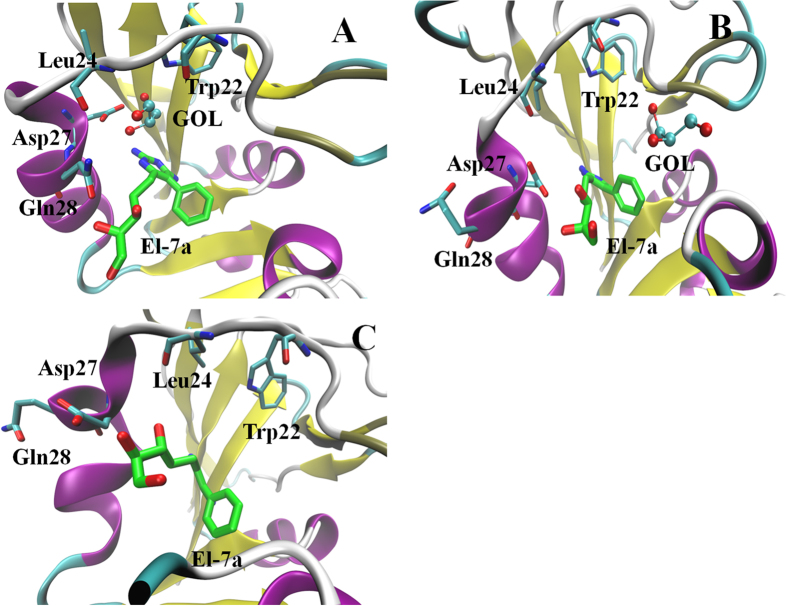
(**A**) The binding poses of compound **El-7a** and GOL at the beginning of simulation. (**B**) at 18 ns and (**C**) at 40 ns. To simplify the figures, NADPH is hidden.

A simulation was performed on the system of *mt*-DHFR in complex with NADPH, GOL and **El-7a**. As shown in Fig. 6A, at the beginning of the simulation, GOL had strong interactions with its binding pocket (Trp22, Leu24, Asp27 and Glu28). With the simulations, compound **El-7a** slowly changed its orientation; the propanetriol group of compound **El-7a** moved towards the GOL binding site. Asp27 gradually moved close to the propanetriol group of compound **El-7a** and formed hydrogen bonds between them. During this process, GOL was slowly expelled from the binding site and left the protein complex (Fig. 6B). At the end of simulation, GOL moved out of the protein completely, and the propanetriol group of compound **El-7a** moved to the GOL binding site and formed hydrogen bonds with Asp27 and Leu24. The results of these simulations suggest that the GOL binding site could be a potential drug target. It must be addressed at here that the GOL is purely a crystallisation artifact, as *mt*-DHFR is usually stored with glycerol to maintain its activity. Therefore, GOL may affect the *in vitro* enzyme assays, but it unlikely can be a necessary co-factor for the *mt*-DHFR in a biologically relevant situation. The simulation with GOL was performed only for the reason to confirm **El-7a** has a strong ability to bind to GOL binding site, even when the binding site has been occupied by GOL.

#### Binding pose analyses of compounds 1–8

**Figure 7:**
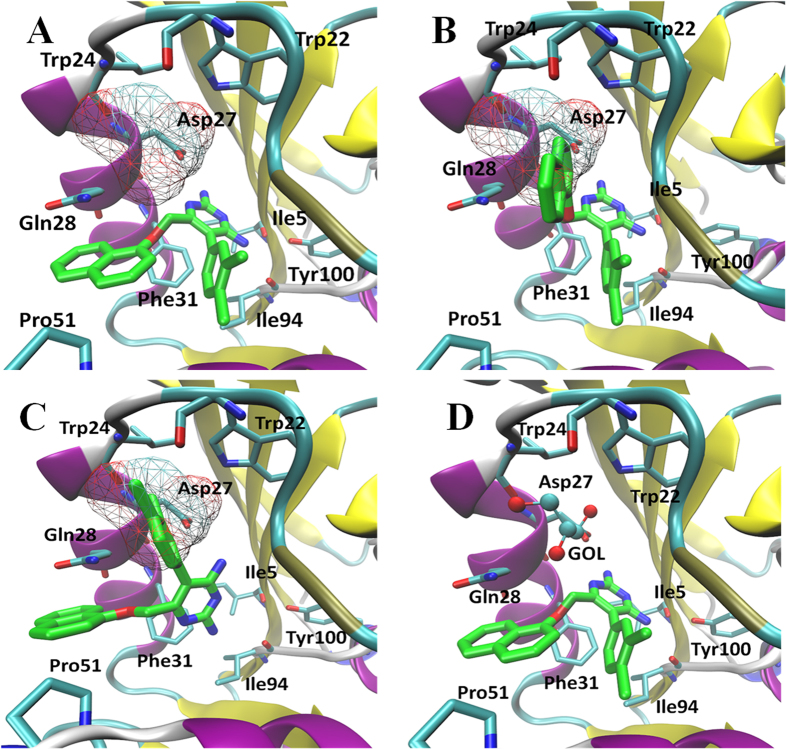
The binding poses of compound **2**. (**A**) Pose 1; (**B**) Pose 2; (**C**) Pose 3; (**D**) Pose 4.

Based on previous simulation of compound **El-7a**, we asked whether compounds **1-8** identified in the present study could bind into the GOL binding site. By comparing the chemical structures of compounds **1–8** with compound **El-7a**, it was observed that only compounds **1**, **2**, **3** and **4** might cause a similar effect as compound **El-7a** because they contain side chains at **6** position on the pyrimidine-2,4-diamines. However, the side chain of compound **4** is much larger than the GOL binding site, which significantly reduces such a possibility. All eight compounds were docked again by GOLD in the absence of GOL and ten binding poses were saved for analysis. As we assumed, only compounds **1-3**, were observed docking with dual binding poses. Among these three compounds, compound **2** showed the strongest inhibition effects on *mt*-DHFR, so it was selected for molecular dynamic simulations for more accurate binding pose analysis. In absence of GOL, compound **2** can be docked with three binding poses: Pose 1 (Fig. 7A), no side group binding to GOL binding site; Pose 2 (Fig. 7B), naphthalene ring binding to GOL binding site; and Pose3 (Fig. 7C), benzene ring binding to GOL binding site. In the presence of GOL, only one binding pose is available, which is similar to the pose 1, and we refer to this as Pose 4 (Fig. 7D). Molecular dynamic simulations, MM-PBSA and Normal Mode calculations were performed on these four binding poses for more detailed conformational and energy analysis.

Through the conformational analysis, we anticipated compound **2** cannot bind into the GOL binding site, because either the naphthalene ring or benzene ring is hydrophobic and cannot have strong interactions with those hydrophilic residues at the GOL binding site. As shown in Table 4, in the absence of GOL, the binding pose 1 showed the lowest binding free energy in comparison with Pose 2 and Pose 3, which may indict compound **2** cannot occupy the GOL binding site, although the energy difference is not very significant. The free energy decomposition showed that in poses 2 and 3, although the naphthalene ring or benzene ring occupy the GOL binding site respectively, none of the key residues (Trp22, Leu24, Asp27 and Glu28) in the GOL binding site have significant free energy contributions (greater than −1 Kcal/mol) for ligand binding. In the presence of GOL, the binding pose 4 shows a similar binding free energy with the consideration of entropy in comparison with the binding pose 1 in the absence of GOL. In binding pose 4, there are strong VDW interactions between GOL and compound **2**, and the total free energy contribution is −1.37 Kcal/mol, which is the 4th strongest contributions among all the residues, and GOL itself has a strong interaction with Trp22 (free energy contribution −2.07 Kcal/mol). The results suggest that GOL functions as a bridge to link compound **2** and *mt*-DHFR, and by this way GOL enhances the enthalpy term of the binding free energy of the compound **2** in *in vitro* enzyme assays, but it also slightly increases the entropy penalties, so that the final binding free energies of compound 2 in pose 1 and pose 4 are almost same. However, in the real biological situation as per the antibacterial activity assays, GOL is unlikely to be a cofactor binding in the GOL binding site, so that binding Pose 1 may be the favourable binding pose in this case. Most DHFR inhibitors target the dihydrofolate binding site. The GOL binding site is a relatively small pocket that is very close to the dihydrofolate binding site and may be treated as an extension of the dihydrofolate binding site. The dihydrofolate binding site is relative large, so although those compounds with large hydrophobic side chains, such as compound 2, cannot occupy the GOL binding site, it can still fit into the dihydrofolate binding site. Once they bind into this site, the reduction of dihydrofolate to tetrahydrofolate can be prevented leading to the inhibition of Mtb growth.

#### The binding pose of compound 6

**Figure 8:**
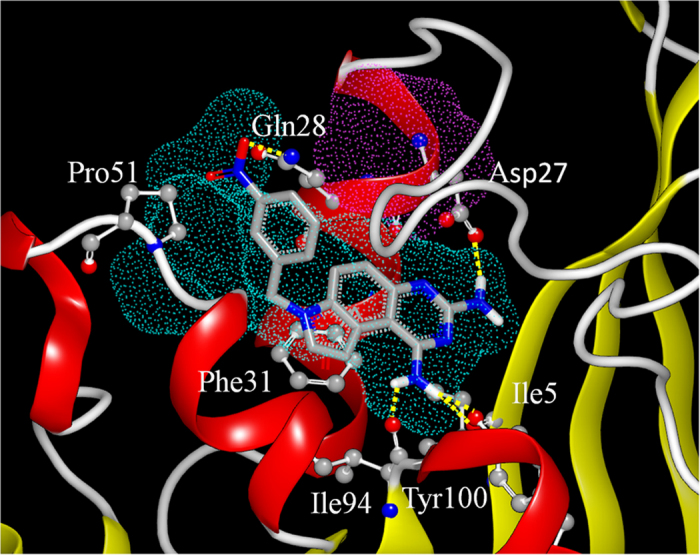
The binding pose of compound 6 in the absence of GOL.

In the present study, as compound **6** was the most potent inhibitor of *mt*-DHFR and exhibited the strongest bactericidal activity, we analysed its binding pose in more detail. Compound **6** was docked in the presence and absence of GOL and this showed no significant differences between these two binding poses. The binding pose of compound **6** is similar to the bound MTX in the crystal structure. Figure 8 shows the results of the docking study in the absence of GOL, with the blue dotted area representing the binding site of MTX (or dihydrofolate) and the purple dotted indicating the binding site of GOL. As predicted, compound **6** occupies the MTX binding site without touching GOL binding site. In this binding pose, it can be observed from the docking results that compound **6** formed 5 hydrogen bonds with Gln28, Asp27, Ile5, Tyr100 and Ile94, and it also formed strong hydrophobic interaction with Pro51 and Phe31.

**Figure 9:**
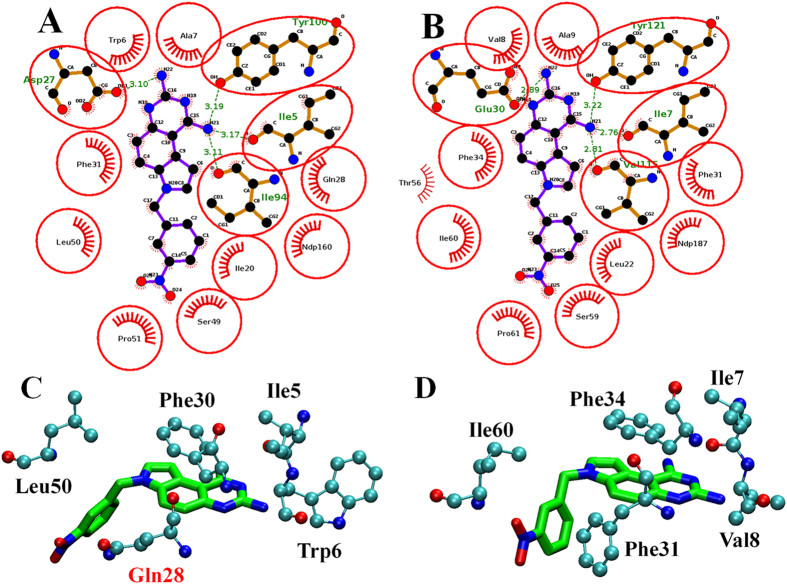
The binding poses and interactions of compound 6 with (**A**) *mt*-DHFR and (**A**) *h*-DHFR. The key interactions (free energy contribution lower than –1 Kcal/mol) between compound 6 and (**C**) *mt*-DHFR, and (**D**) *h*-DHFR.

The binding poses of compound **6** bound with *mt*-DHFR and *h*-DHFR were compared. It was observed that the binding pockets of these two proteins are highly conserved, and the binding poses of compound **6** are almost identical (Fig. 9), which is consistent with the fact that compound **6** only shows a very slight degree of selectivity against *h*-DHFR. DHFR inhibitors usually contain a central core of 2,4-diaminopteridin (classical inhibitors) or pyrimidine-2,4-diamines (non-classical inhibitors). However, compound **6** contains a novel central core (7H-pyrrolo[3,2-f]quinazoline-1,3-diamine), which will significantly expand the chemical space of novel DHFR inhibitors.

Molecular dynamic simulations were performed on compound 6 in complex with *h*-DHFR and *mt*-DHFR in the absence of bound GOL, as GOL is not a cofactor in the real biological situation. Through the conformation analyses along both trajectories, compound 6 was tightly bound to both proteins, and the binding poses were stable along the entire simulations. The MM-PBSA and Normal Mode calculations were performed on both systems, and it was found that the binding free energies of these two simulations were similar (Table 5), but the one with *h*-DHFR is slightly lower than that of *mt*-DHFR (only about −0.76 Kcal/mol), which does not fully match the experimental data. The reason for this is likely to be a result of system error, because such small differences in binding affinities cannot be accurately predicted by MM-PBSA and Normal Mode calculations, especially when the receptors are two different proteins. We also observed that the binding free energy of compound 2 with *mt*-DHFR is lower than that of compound 6, which contradicts the experimental data, most likely because the MM-PBSA and Normal Mode approaches are better at selecting poses for one compound than ranking molecules with significantly different structures (which is certainly true for compounds 2 and 6).

A free energy decomposition of MM-PBSA calculation was performed to analyze the interaction of compound 6 with both *mt*-DHFR and *h*-DHFR. We observed that 4 residues contributed strongly to the binding free energy of compound 6 with *mt*-DHFR (lower than −1.0 Kcal/mol). They are Ile5 (−1.56 Kcal/mol), Trp6 (−1.61 Kcal/mol), Phe31 (−2.32 Kcal/mol) and Leu50(−1.02 Kcal/mol). Among these residues, Ile5 (−1.35 Kcal/mol VDW, −1.73 Kcal/mol electrostatic interactions) and Trp6 (−1.19 Kcal/mol VDW, −0.94 Kcal/mol electrostatic interactions) show both VDW and electrostatic interactions, and Phe31 and Leu50 mainly show strong VDW interaction. In the interactions between *h*-DHFR and compound 6, there are five residues (Ile7 (−1.74 Kcal/mol), Val8 (−1.54 Kcal/mol), Phe31 (−1.10 Kcal/mol), Phe34 (−2.34 Kcal/mol) and Ile60 (−1.50 Kcal/mol) that demonstrated strong free energy contributions (lower than −1.0 Kcal/mol). Among them, only Ile7 show free energy contributions from both VDW (−1.33 Kcal/mol) and electrostatic interactions (−1.72 Kcal/mol), and the remaining residues mainly contribute to the binding free energy via VDW interactions. The hydrophobic residue Phe31 in h-DHFR showed a strong free energy contribution, but the corresponding residue in *Mt*-DHFR, Gln28 (with a polar side chain, labeled in red in Fig. 9c), only showed a weak free energy contribution (−0.37 Kcal/mol). This difference could be a potential direction for future molecular design attempts to improve the selectivity of compound 6 analogues, for example by adding more polar groups at the corresponding position. Another more obvious direction is the addition of a GOL like group on compound 6 to occupy the GOL binding site.

## Conclusion

Using *in silico* methods, we have identified a set of *mt*-DHFR inhibitors that inhibited recombinant *mt*-DHFR and *h*-DHFR to varying degrees and that showed bactericidal effects against *M. tuberculosis* H37Ra. Molecular simulations indicated that the GOL binding site can be occupied by inhibitors containing appropriate side chains. Among the compounds identified in the present study, only the compounds **1**, **2**, **3** and **4** have such possibility. However, they all contain large hydrophobic side chains at 6 position on the pyrimidine-2,4-diamines. As the GOL binding site is more hydrophilic, and contains hydrogen bond acceptors and donors, these compounds failed to occupy the GOL site, which could be one of the reasons that the present inhibitors did not show strong selectivity against *h*-DHFR. Compound **6** was the most potent inhibitor obtained in the present study. It contains a novel central core (7H-pyrrolo[3,2-f]quinazoline-1,3-diamine), which will significantly expand the chemical space of novel DHFR inhibitors. This compound did show a small degree of selectivity against *h*-DHFR and can be used as a lead compound for further optimization and will inform future medicinal chemistry efforts to improve the selectivity of compounds against *mt*-DHFR.

Whilst the GOL binding site is not essential for *mt*-DHFR enzyme activity, it is thought to be important for the selectivity for *mt*-DHFR against *h*-DHFR. Our modelling study suggests hydrophilic side groups may be able to occupy this binding site. This approach may lead to new inhibitors being discovered that specifically bind to *mt*-DHFR. However, it is cautionary to note that as Mtb has a thick cell wall that only allows highly hydrophobic compounds to pass through, adding a hydrophilic side group to compounds may prevent them from crossing the mycobacterial cell wall. Consequently, although such compounds may be able to specifically inhibit *mt*-DHFR, they may not be able to act on Mtb whole cells. Therefore, finding a balance between selectivity and activity on Mtb remains a major challenge in anti-TB drug discovery.

## Additional Information

**How to cite this article**: Hong, W. *et al*. The identification of novel Mycobacterium tuberculosis DHFR inhibitors and the investigation of their binding preferences by using molecular modelling. *Sci. Rep*. **5**, 15328; doi: 10.1038/srep15328 (2015).

## Figures and Tables

**Figure 1:**
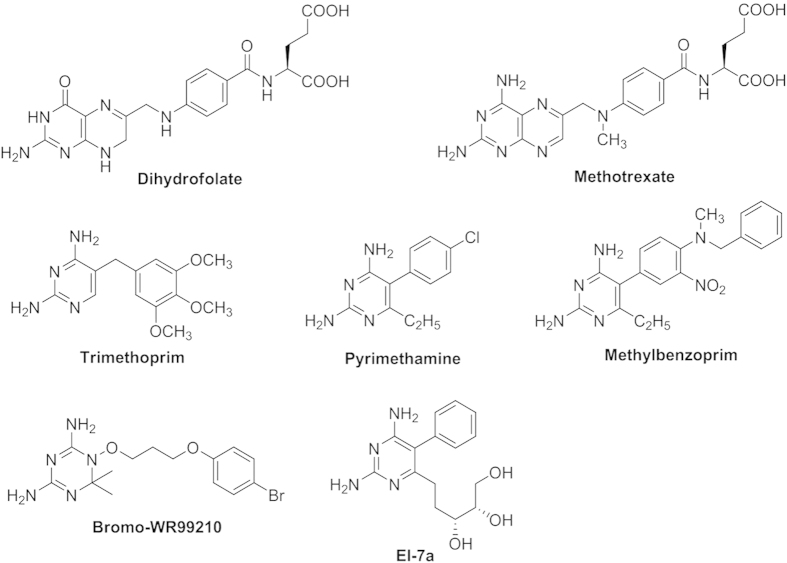
The known inhibitors of mt-DHFR.

**Figure 2:**
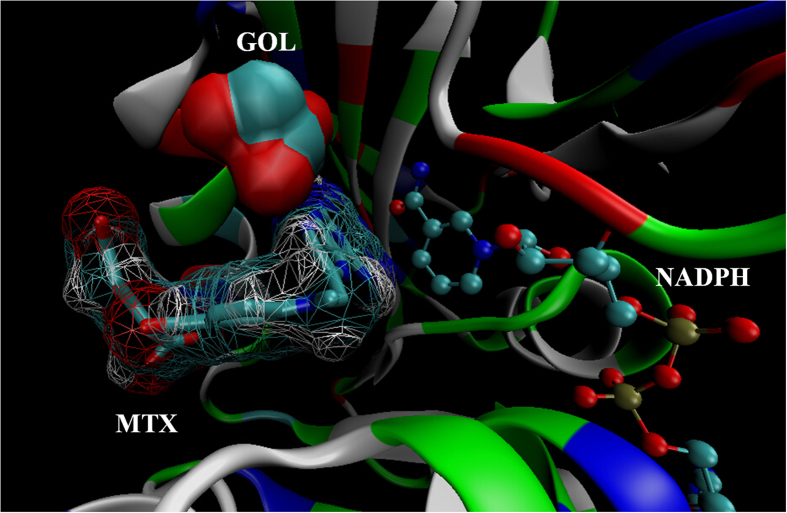
The binding sites of MTX, GOL and NADPH in the crystal structure of *mt*-DHFR.

**Table 1:**
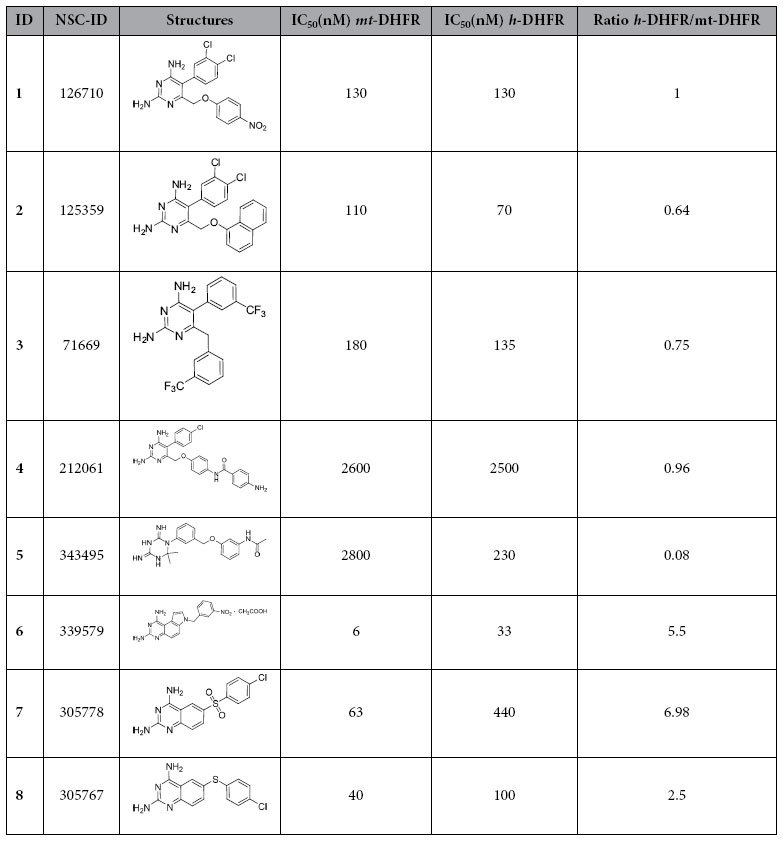
Structures and activity against recombinant *mt*- and *h*-DHFR.

**Table 2:** Anti-mycobacterial activity of compounds **1**–**8** against Mtb H37Ra, showing colony counts after four weeks of exposure to each compound.

Compound	Compound concentration (μg/ml)					
	100	50	10	5	1	0.1
**1**	+++	+++	+++	+++	+++	+++
**2**	++	++	+++	+++	+++	+++
**3**	+	++	+++	+++	+++	+++
**4**	—	++	+++	+++	+++	+++
**5**	+++	+++	+++	+++	+++	+++
**6**	—	—	++	+++	+++	+++
**7**	+++	+++	+++	+++	+++	+++
**8**	—	+++	+++	+++	+++	+++
PAS (4 μg/ml)	++					

−no growth; +, 1–10 colonies; ++, 11–100 colonies; +++, colonies too numerous to count.

**Table 3:** Binding free energies (Kcal/mol) of compound **El-7a** in Pose 1 and 2.

Simulationns	∆E_vdw_	∆E_ele_	∆G_pb_	∆G_np_	∆G_gas_	∆G_solv_	∆G_mmpbsa_	T∆S	∆G_binding_
El-7a (Pose 1)	−41.28 ± 0.07	−43.35 ± 0.14	59.64 ± 0.12	−4.76 ± 0.003	−84.63 ± 0.14	54.89 ± 0.12	−29.74 ± 0.09	−23.26 ± 1.20	−6.48
El-7a (Pose 2)	−39.41 ± 0.06	−15.80 ± 0.13	34.80 ± 0.11	−5.22 ± 0.003	−55.21 ± 0.13	29.59 ± 0.11	−25.62 ± 0.07	−22.05 ± 1.2	−3.57

**Table 4:** Binding free energies (Kcal/mol) of compound **2** in Poses 1, 2, 3 and 4.

Compound 2	∆E_vdw_	∆E_ele_	∆G_pb_	∆G_np_	∆G_gas_	∆G_solv_	∆G_mmpbsa_	T∆S	∆G_binding_
Pose 1	−47.77 ± 0.09	−15.62 ± 0.09	37.41 ± 0.13	−5.43 ± 0.005	−63.39 ± 0.12	31.98 ± 0.13	−31.41 ± 0.11	−21.45 ± 1.46	−9.96
Pose 2	−48.05 ± 0.06	−14.50 ± 0.07	37.51 ± 0.09	−5.54 ± 0.003	−62.55 ± 0.08	31.97 ± 0.09	−30.58 ± 0.09	−22.07 ± 1.08	−8.51
Pose 3	−45.47 ± 0.10	−21.28 ± 0.08	42.98 ± 0.18	−5.56 ± 0.004	−66.75 ± 0.15	37.41 ± 0.18	−29.34 ± 0.10	−21.79 ± 1.24	−7.55
Pose 4	−48.22 ± 0.07	−11.89 ± 0.07	32.33 ± 0.08	−5.34 ± 0.005	−60.11 ± 0.09	26.99 ± 0.08	−33.13 ± 0.08	−23.55 ± 1.05	−9.58

**Table 5:** Binding free energies (Kcal/mol) of compound **6** in mt-DHFR and h-DHFR.

Compound 6	∆E_vdw_	∆E_ele_	∆G_pb_	∆G_np_	∆G_gas_	∆G_solv_	∆G_-mmpbsa_	T∆S	∆G_binding_
*mt*-DHFR	−45.69 ± 0.05	−14.05 ± 0.08	35.72 ± 0.09	−5.18 ± 0.003	−59.73 ± 0.10	30.54 ± 0.09	−29.19 ± 0.07	−23.55 ± 1.23	−5.64
*h*-DHFR	−46.76 ± 0.06	−15.34 ± 0.10	38.64 ± 0.11	−5.11 ± 0.002	−62.10 ± 0.11	33.52 ± 0.11	−28.58 ± 0.08	−22.18 ± 1.20	−6.40
